# Syndecan-1: A Review on Its Role in Heart Failure and Chronic Liver Disease Patients' Assessment

**DOI:** 10.1155/2019/4750580

**Published:** 2019-11-11

**Authors:** Radu-Stefan Miftode, Ionela-Lăcrămioara Şerban, Amalia-Stefana Timpau, Ionela-Larisa Miftode, Adriana Ion, Ana-Maria Buburuz, Alexandru-Dan Costache, Irina-Iuliana Costache

**Affiliations:** ^1^Department of Internal Medicine I (Cardiology), Faculty of Medicine, University of Medicine and Pharmacy “Gr. T. Popa”, Iasi 700115, Romania; ^2^Department of Morpho-Functional Sciences (II), Faculty of Medicine, University of Medicine and Pharmacy “Gr. T. Popa”, Iasi 700115, Romania; ^3^Department of Infectious Diseases, Faculty of Medicine, University of Medicine and Pharmacy “Gr. T. Popa”, Iasi 700115, Romania

## Abstract

The close connection and interaction between the cardiac and the liver functions are well-known, as cirrhotic cardiomyopathy is an important clinical entity which best describes the mutual pathogenical influence between these two organs. Due to the fact that cardiac dysfunction in patients with chronic hepatic disorders is oligosymptomatic or even asymptomatic, an early diagnosis represents a challenge for every physician. Syndecan-1—a transmembrane proteoglycan that exerts its functions mainly via its heparane sulfate chains—is a very promising biomarker, correlated not only with the degree of cardiac fibrosis but also with the severity of liver fibrosis. Many studies highlighted its role in the development of cardiac fibrosis or atherogenesis, being significantly correlated with the activity of angiotensin II. Multiple evidence revealed that syndecan-1 is also associated with tissue injury and may regulate inflammatory and regenerative responses, being considered a protective molecule that limits the inflammation and reduces cardiac remodelling and dysfunction after a myocardial infarction. Syndecan-1 may also be used as a reliable biomarker for the noninvasive assessment of liver fibrosis. Under various fibrogenetic conditions, shedding of syndecan's extracellular domain took place, becoming a soluble form that binds different growth factors and inhibits further fibrosis. This complex molecule is also involved in the lipid metabolism, by altering the clearance of cholesterol particles, and in chronic hepatitis, by enhancing the viral invasion of hepatocytes. Due to the growing interest in this biomarker, multiple studies aimed at revealing syndecan-1's potential benefits in the diagnosis and prognosis assessment in patients with heart failure or chronic liver disorders. In this review, we review the mechanisms by which syndecan-1 exerts its effects and the possible perspectives opened by its use as a dual cardio-hepatic biomarker.

## 1. Introduction: The Influence of Cirrhosis on the Cardiovascular System

The most important hemodynamic feature in cirrhotic patients is the hyperdynamic circulatory status, characterized by an increased cardiac output, low blood pressure, and decreased vascular resistance. The cardiac consequence of this hemodynamic profile is represented by the cirrhotic cardiomyopathy (CCM), a clinical entity comprising systolic and diastolic dysfunctions and numerous electrophysiological abnormalities, in the absence of any other heart diseases [1, 2].

Basically, the pathogenesis of CCM involves cellular and neurohumoral factors that can determine alterations of cardiomyocytes' cellular membrane, resulting in modifications in calcium signalling, exaggerated stimulation of beta receptors, and abnormal levels of endocannabinoids. In cirrhotic patients, there is also an increase in circulating levels of vasoactive substances (e.g., endothelin, tumor necrosis factor, prostacyclins, and nitric oxide), of which the toxic effect of nitric oxide is of high importance. [3, 4].

Because CCM is oligosymptomatic or even asymptomatic, an early diagnosis represents a challenge for every physician, while its real prevalence is very limited. Diastolic dysfunction (due to myocardial fibrosis, hypertrophy of cardiomyocytes, and subendothelial edema) occurs before systolic impairment, which is generally observed in situations when an imbalance occurs between an increased demand for cardiac output and an impaired myocardial contractility (e.g., infections, surgery and intense physical exercises) [3, 5].

Thus, the research for a biomarker able to detect a subclinical heart failure at an early stage in cirrhotic patients is of high importance. Syndecan-1 is a very promising biomarker, being correlated not only with the degree of cardiac fibrosis [6] but also with the severity of liver fibrosis and long-term risk of hepatic carcinoma [7, 8]. In this paper, we aim to evaluate some perspectives on syndecan-1's role in the study of heart failure in patients with chronic liver disease.

## 2. Syndecan-1: Structural and Biochemical Characteristics

Syndecan-1 is a subtype of the vast transmembrane proteoglycans (PGs), which are composed of a core protein to which growth factor binding glycosaminoglycan (GAG) side chains are attached. The syndecan family consists of four members, located in various tissues, only syndecan-4 presenting a ubiquitous expression [9].

The core protein of syndecans (20–40 kDa) consists of a highly conserved C-terminal cytoplasmic domain, a single-pass transmembrane domain, and a large N-terminal extracellular domain, this ectodomain being the attachment site for up to five GAG chains [9, 10]. Also, it is worth mentioning that syndecan-1 from different tissues presents different GAG types comprising heparan sulfate (HS) and chondroitin sulfate (CS) of varying length and ultrastructure.

The biochemical reactions that modify the basic structure of HS represent a way to regulate the protein binding PGs [9] because syndecan-1's biological importance is directly determined by its capacity to bind a plethora of ligands via their HS chains, as there is more evidence indicating that they have roles in matrix interactions and, sometimes, even in matrix assembly [10].

In contrast with the ectodomain, Couchman highlighted since 2003 that the transmembrane region and the cytoplasmatic domains of syndecans are highly conserved, requiring multiple interactions in order to be capable of intercellular signalling [11].

Several studies revealed that the shedding of the ectodomain (mediated by matrix metalloproteinases) is another important structural aspect through which syndecan influences the molecular processes, converting the cell-attached syndecan to a soluble active ligand. The latter is explained by the presence of intact GAG chains on the released ectodomain; thus, the soluble syndecan-1 has preserved its ability to modulate biological processes (e.g., growth factor responses), as shown in the ample analysis of Szatmari [9].

Other experimental studies conducted by Nikolova and Teng have highlighted that membrane-bound and soluble forms of syndecan-1 may even have opposing effects, especially in carcinogenesis. This is the result of binding soluble syndecan-1 to proangiogenic factors like VEGF or FGF, a process which activates them, creating a so-called chemotactic gradient, promoting invasion of endothelial cells and angiogenesis [12, 13]. In contrast, through TGF*β*1-binding, thus enhancing its clearance, overexpression of soluble syndecan-1 is highly associated with fibrosis, either myocardial or hepatic, extensive evidence being provided by several studies [6, 7, 14, 15].

## 3. Syndecan-1 in Hypertensive Heart Failure: The Renin-Angiotensin-Aldosterone System

The profibrotic role of angiotensin II (Ang II) in cardiac remodelling is a well-known process mediated by the concomitant activation of the transforming growth factor *β*-1 (TGF*β*1) and the connective tissue growth factor (CTGF) via cell signalling, thus leading to an enhanced synthesis of collagen and other multiple matrix proteins [16, 17]. By promoting these structural alterations, Ang II is causing myocardial hypertrophy and, subsequently, fibrosis, an effect that is doubled by its potent vasoconstrictor capacity.

Like syndecans, Ang II-mediated signalling occurs mainly via heparin sulfate chains [18], by their ability to bind to the extracellular matrix (ECM). This bond will further stimulate the production of not only growth factors but also their receptors, such as endoglin, a TGF*β*1 receptor. A possible blockade of this signalling pathway arises as an option for decreasing Ang II-induced myocardial fibrosis [16, 17].

Based on these previous observations and the assumed role of proteoglycans in cardiac fibrosis, Schellings et al. highlighted some interesting results regarding the involvement of syndecan-1 in cardiac remodelling by affecting growth factor-mediated signalling, hence revealing that the absence of syndecan-1 in mice protects against Ang II-induced cardiac dysfunction and subsequent fibrosis [19]. These findings are supported by the significantly increased syndecan-1 expression in Ang II-treated mice, predominantly in areas with fibrotic myocardium. The same authors identified an increased *in vivo* expression of collagen I and III and connective tissue growth factor (CTGF) in mice cardiac fibroblasts, after an infusion with Ang II. Also, they observed that the syndecan-1 null mice are protected against Ang II-induced cardiac dysfunction or fibrosis, because the loss of syndecan-1 attenuates the effect of Ang II, by reducing the induction of growth factors (CTGF, TGF*β*1).

Another important aspect is represented by the involvement of HS chains in fibrogenic signal transduction. The addition of protamine (an inhibitor of heparin and heparan sulfate) prevented the increase in CTGF and collagen I expression after Ang II stimulation. In addition, cardiac fibroblasts treated with syndecan-1 ectodomain, but without HS chains, had no effect on CTGF expression, furtherly confirming that heparan sulfates are involved in syndecan-1's fibrogenic signal transduction [19, 20].

## 4. Syndecan-1 in Ischemic Heart Failure: Its Importance in the Evolution of Atherosclerosis

Several studies assessed syndecan-1 expression in atherogenesis and the degree to which Ang II influences syndecan-1 expression in macrophages from atherosclerotic lesions. Substantial evidence supports the theory that Ang II may exert significant proatherogenic effects within the vascular wall. For example, Ang II enhances the activity of macrophage lipoxygenase with subsequent generation of proatherogenic oxidized LDL, thereby accelerating parietal monocyte recruitment [21]. Another incriminated mechanism is the Ang II-induced expression of macrophage HMG CoA reductase, which determines increased synthesis of cholesterol with important uptake of oxidized LDL and subsequent development of foam cells [22, 23].

Wang et al. observed that syndecan-1 was highly expressed in macrophages from atherosclerotic lesions induced after an 8-week infusion with Ang II in ApoE-deficient mice [24]. The same authors also revealed that Ang II upregulates the syndecan-1 expression via MAP kinase signalling, and these kinases are being activated either directly by AT1 receptor or via reactive oxygen species and hydrogen peroxide produced by membrane NAD(P)H oxidases in response to Ang II. In addition, Ang II is a potent inducer of syndecan-1 shedding into the ECM, a process that could accelerate atherosclerotic lesion formation by enhancing the generation of a cellular environment with a marked proinflammatory and growth-stimulating status. This occurs because shed syndecans are capable to interact with a variety of heparin-binding proinflammatory chemokines (e.g., monocyte chemoattractant protein-1), via their HS chains, therefore potentiating wall lesions in the form of atherosclerotic plaque.

More important, in a recent study, Vo revealed that syndecan-1 is markedly expressed even in early atheromatous lesions of aorta, such as fatty streaks and fibrolipidic lesions [25]. Syndecan-1 is found particularly on intimal smooth muscle cells, favouring the accumulation of foam cells.

However, other authors found a protective role of syndecan-1 in atherosclerosis, based mainly on its well-established anti-inflammatory functions. There is increased evidence that syndecan-1 expression is associated with a high intrinsic motility of macrophages, while its deficiency is characterized by the impaired migration and increased adhesion to ECM. The assessment of wild type, syndecan-1 positive mice compared with syndecan-1 deficient mice highlighted an enhanced density of inflammatory macrophages, resulting in a greater risk of atherosclerotic plaque for the latter [26, 27].

## 5. Syndecan-1 in Acute Myocardial Infarction: Protective vs Controversial Effects

Extensive evidence revealed that syndecan-1 is associated with tissue injury and may regulate inflammatory and regenerative responses. However, these mechanisms are highly variable and context-dependent. After assessing syndecan-1 serum levels following a myocardial infarction in mice, Vanhoutte et al. observed that syndecan-1 is an essential protective molecule that limits the inflammation and reduces myocardial remodelling and dysfunction [28]. These findings are based on its presumed role as an inhibitor of leukocyte-endothelium interaction. In addition, a proinflammatory environment enhances the activity of matrix metalloproteinases, with the “de novo” synthesized collagen being of inferior quality, hence predisposing to cardiac remodelling and dilatation. In another study, it was hypothesized that these effects may also be mediated by regulation of TGF*β* signalling by syndecan-1, with an enhanced anti-inflammatory response in myocardial infarction, in contrast to the profibrotic action exhibited in Ang II-treated hearts [29].

Other research studies highlighted that in acute myocardial infarction, there are elevated serum levels of syndecan-1, due to an increased sympatho-adrenergic activation and subsequent damage to endothelial glycocalyx. The strongest association was found between the blood levels of syndecan-1 and adrenaline, both were independently associated with long-term mortality in patients with cardiogenic shock following infarction [29]. In a recent study, Fuernau et al. also reported a significant association between high syndecan-1 levels and short-term mortality in 600 patients with infarct-related cardiogenic shock, furtherly confirming previous data [30].

## 6. Syndecan-1: A Valuable Marker of Liver Fibrosis?

Even though biomarkers for noninvasive assessment of liver fibrosis have been widely tested, there are scarce data about the utility of syndecan-1 in the evaluation of cirrhotic patients. The chains of syndecan-1 are binding several molecules, facilitating intercellular signalling, the most important interactions in liver fibrosis being the ones with TGF*β*1, basic fibroblast growth factor (bFGF), and hepatocyte growth factor (HGF) [31].

An important feature of syndecan-1 molecule is the shedding of the extracellular domain, which occurs in various pathological conditions, thus transforming the ectodomain in a soluble form, under the influence of several proteolytic enzymes. The blood concentration of this soluble form composed of shed extracellular domain increases in liver diseases such as nonalcoholic fatty liver disease or liver fibrosis and is reported as a potentially useful biomarker for disease monitoring [32].

Data from a recent study indicate that syndecan-1 overexpression inhibits liver fibrogenesis through its shedding. These shed ectodomains of syndecan-1 bind and enhance TGF*β*1 clearance, one of the most important promotors of fibrogenesis [14]. In addition, it was observed that syndecan-1 can bind thrombospondin-1, an activator of TGF*β*1 and another pathway to prevent fibrogenesis. An additional aspect refers to syndecan-1's capacity to trigger the synthesis of matrix metalloproteinase (MMP), especially MMP-14, which is critically involved in ECM degradation, therefore hampering the progression of fibrosis. However, the protective effect exerted by syndecan-1 lasts only for 2 to 4 months, and then immunochemistry detects low levels of HS chains, suggesting an exhausted synthesis of syndecan-1 [14].

## 7. The Involvement of Syndecan-1 in Different Chronic Liver Disorders

Several studies have shown that an increased syndecan-1 expression may be associated with different chronic liver diseases. The utility of serum syndecan-1 as a reliable, noninvasive biomarker for liver fibrosis was highlighted by Zvibel et al. in patients with chronic hepatitis C [7]. In addition to these data, Metwaly et al. have observed that elevated serum levels of syndecan-1 are significantly correlated with the presence of hepatocellular carcinoma compared not only with healthy controls, but also with cirrhotic patients [33]. A more recent research from Baghy et al. [34] revealed only a rather small increase in syndecan-1 expression in cancer without cirrhosis, compared with cancer with cirrhosis which, in contrast, presented a significantly higher expression of syndecan-1.

Regarding the nonalcoholic fatty liver disease (NAFLD), it is worth mentioning that levels of syndecan-1 are significantly higher in patients with biopsy-confirmed NAFLD compared with controls with normal liver [35]. Nevertheless, these findings were not supported by a correlation between NAFLD-associated fibrosis and the levels of syndecan-1, as in the case of fibrosis due to hepatitis C. A possible explanation for this discrepancy is represented by the presence of different risk factors for hepatitis C-induced fibrosis (insulin resistance and virus-induced liver inflammation), compared with the factors that facilitate fibrosis in patients with NAFLD (steatosis and systemic inflammation).

However, in the case of patients with chronic liver diseases and concomitant clostridium colitis, with a significant inflammatory status, the use of syndecan-1 as a reliable liver fibrosis biomarker is marked by controversy because of its important variations in inflammatory diseases [13, 36].

## 8. Syndecan-1 Enhances Viral Invasion of Hepatocytes

The strong link between syndecan-1 and hepatitis C was also questioned at molecular level by Grigorov et al., as hepatitis C virus (HCV) infects hepatocytes after binding to heparan sulfate chains, largely present on syndecan-1 [37]. The same researchers observed that viral internalization occurs via direct interaction between syndecan-1 and CD81 at the basolateral level of the hepatocyte membrane. This finding was supported by the observation that the blocking of syndecan-1 inhibited HCV infection, while the knocking-down of both syndecan-1 and CD81 exerted an even more potent inhibitory action upon viral internalization.

## 9. Syndecan-1 and Liver Cancer

A very recent study from Regős et al. assessed the expression of syndecan-1 in liver cancer, observing that most important augmentation of syndecan-1 expression was found in cirrhosis due virus C infection and in its most severe complication, the hepatocellular carcinoma (HCC). The authors also noticed that even syndecan-1 levels are increased both in HCC with or without previous cirrhosis, its expression being significantly higher in the former, with high levels also detected in the peritumoral cirrhotic areas [38].

Concerning the correlation between syndecan-1 levels and the tumor stage, a parallel increase was observed, but without reaching the threshold of statistical significance.

## 10. Syndecan-1 and Its Critical Role in Lipid Clearance

Another particularity of the syndecan-1 molecule, related to lipid metabolism, was observed by Deng et al., who demonstrated that membrane syndecan-1 can mediate the binding and uptake of the very low-density lipoprotein (VLDL) [39]. In various conditions, such as inflammation, ischemia, or under the influence of certain growth factors or bacterial components, there is an increased shedding of syndecan-1 from the hepatic cells, via the activation of multiple metalloproteinases. These syndecan‐1 ectodomains bind VLDL in the space of Disse and prevent their clearance, resulting in an impaired VLDL catabolism with subsequent hypertriglyceridemia.

## 11. Possible Diagnosis and Prognosis Role of Syndecan-1 in Patients with Heart Failure and Concomitant Liver Diseases

Cardiovascular comorbidities in liver disease represent any simultaneous pathological findings that are neither causes nor consequences of cirrhosis, but may increase the mortality and have a negative influence on the therapeutic outcome.

Syndecan-1, as an emerging biomarker for both heart failure and liver fibrosis, could find its usefulness either in the initial assessment or the long-term prognosis in a large number of patients with concomitant cardiac and hepatic diseases. This potential role as a dual cardio-hepatic biomarker is based on several findings highlighting a very high prevalence of cardiovascular diseases among cirrhotic patients [40, 41].

Generally, cirrhotic patients with heart failure have a preserved left ventricular ejection fraction (EF > 50%), with the diastolic dysfunction being dominant, due to myocardial hypertrophy (induced by a hyperdynamic circulation) and subsequent fibrosis with cardiac stiffness that alter the left ventricular filling pressures. In these patients, with preserved EF, where echocardiographic assessment for heart failure is equivocal, syndecan-1 could represent an early diagnosis marker or it could, at least, guide the clinician towards more advanced explorations if its serum levels are above cutoff values.

Cirrhotic patients with impaired EF (EF < 40%) are vastly diagnosed with dilated cardiomyopathy, this being also significantly associated with a toxic etiology of cirrhosis. This particular situation is predominantly revealed in situations with an increased demand for cardiac output associated with impaired myocardial contractility, such as in conditions of hemodynamic stress infections, physical effort, certain medication, and surgery (especially liver transplant) as shown in a study conducted by Barbosa et al. [42].

In terms of clinical outcome after a hospitalization for heart failure of various etiologies (ischemic and nonischemic), Tromp et al. observed a strong correlation between syndecan-1 levels and a poor prognosis in patients with heart failure and preserved EF, but not in patients with reduced EF [15]. However, recent findings from Liu et al. revealed that a high serum syndecan-1 is associated with a negative outcome also in patients with reduced EF of nonischemic etiology (e.g., nonischemic dilated cardiomyopathy, frequently met in patients with ethanolic cirrhosis), thus highlighting the syndecan-1's capacity of being a useful prognosis biomarker no matter how well-preserved the EF is [43]. Notably, some data from literature show that higher levels of syndecan-1 were correlated with the symptomatology and severity of heart failure, an elevation of the serum biomarker being more commonly met in patients with NYHA III/IV functional class, compared to those in NYHA I/II [44, 45].

Various studies have previously confirmed an association not only between HCV infection and cardiomyopathy [46, 47] but also between HCV infection and the expression of syndecan-1 [37, 38]. Hence, a routine assessment of syndecan-1 levels in these HCV-infected patients could unmask a subclinical progression towards liver fibrosis or, more often, an early cardiac dysfunction. In a recent study, Poller et al. presented some promising preliminary results regarding the positive cardiac effect exerted by the new interferon-free regimens in patients with advanced heart failure (NYHA III-IV classes) and concomitant chronic hepatitis C, with an improvement of their functional status and NYHA class [48]. Considering these findings, of equal importance, may also be the utilization of syndecan-1 in the monitoring of therapeutic effectiveness in patients with chronic hepatitis C who receive the new interferon-free therapies. Basically, syndecan-1 levels below cutoff values would sustain a “fibrosis-free” HCV-infected patient, both at hepatic and myocardial level.

It is well-known that previously diagnosed hypertensive patients become normotensive during the progression of liver fibrosis, due to vasodilatation with low overall systemic vascular resistance and high arterial compliance. However, high blood pressure was incriminated as an independent risk factor for NAFLD and nonalcoholic steatohepatitis, a direct correlation being already proven in multiple studies [35, 49, 50]. Taken into account that these two clinical entities can progress to liver fibrosis or even cirrhosis, the dynamic evaluation of syndecan-1 serum levels in patients with these chronic disorders seems reasonable.

## 12. Conclusions

This research discusses the multiple pathophysiological roles of syndecan-1, many of them still being poorly understood. There is growing evidence highlighting the importance of syndecan-1 as an emerging biomarker for the diagnosis and prognosis of heart failure and also in the noninvasive assessment of liver fibrosis.

Its role in heart failure due to myocardial ischemia or infarction is not to be neglected, where the protective effects mediated by syndecan-1 are based on its anti-inflammatory properties. However, there is still room to debate as to syndecan-1's precise role in ischemic heart failure, given the fact that it also accelerates atherosclerosis.

Results presented in literature underline the beneficial effect of syndecan-1 in the early stages of liver fibrosis, by way of binding and enhancing the clearance of TGF*β*1, an important promoter of fibrogenesis. In contrast, the syndecan-1 molecules from hepatocyte membrane also facilitate the HCV invasion, determining a long-standing chronic infection with multiple complications.

Finally, taken into consideration the increased prevalence of heart failure in patients with chronic liver diseases and vice-versa, syndecan-1 seems to be an attractive cardiac and hepatic dual biomarker, ready to be used as a diagnosis as well as a prognosis marker. However, the final confirmation for this promising biomarker will be granted by the results of future, large-scale, prospective studies that will include patients with concomitant cardio-hepatic pathology.
